# Views on patient portal use for adolescents in mental health care - a qualitative study

**DOI:** 10.1186/s12913-023-09156-6

**Published:** 2023-02-09

**Authors:** Martine Stecher Hornum, Aslak Steinsbekk, Torunn Hatlen Nøst

**Affiliations:** 1 Department of Mental Health, Faculty of Medicine and Health Sciences, Norwegian University of Science and Technology, 7491 Trondheim, Norway; 2 Department of Public Health and Nursing, Faculty of Medicine and Health Sciences, Norwegian University of Science and Technology, Trondheim, Norway; 3 Norwegian Centre for E-health Research, Tromsø, Norway; 4 Norwegian Advisory Unit on Complex Symptom Disorders, St. Olavs hospital, Trondheim University Hospital, Trondheim, Norway

**Keywords:** Patient portals, Telehealth, eHealth, Electronic health records, Adolescent mental health care

## Abstract

**Background:**

Digitalization of health care has opened up for new ways to involve and engage patients. With this, increased attention has been put on digital patient portals. There exists some research on patient portals for adolescent patients in general and for adults in mental health care. However, no studies on patient portals for adolescents in mental health care have been identified in a recent review. The aim was therefore to explore the views on using patient portals for adolescents in mental health care among persons involved in and/or being affected by the introduction of a patient portal.

**Methods:**

A qualitative study was conducted using individual semi-structured interviews with 14 persons who were healthcare providers in child and adolescent mental health care, young representatives from the user panel, or persons affiliated with an EHR-project introducing a patient portal. The main questions addressed their views on introducing patient portals for adolescents in mental health care and how patient portals and access to clinical notes can affect them and their treatment.

**Results:**

The findings were categorised into four main themes; “Does access to a patient portal help or harm adolescents?”, “Who decides access?”, *“*Mostly a political goal” and “Need for support and competency”. Informants mentioned situations in which both adolescents’ and parents’ access to a patient portal could help adolescents in mental health care, but also where it could potentially harm their treatment and threaten confidentiality. Most informants thought that healthcare providers should have the autonomy to determine which information should be shared with whom, but also requested guidelines to ensure equal practice and support in difficult situations. Some perceived patient portals as the result of a political decision, rather than healthcare providers´ wishes, while others described it as a necessary development towards democratization.

**Conclusion:**

The informants’ views varied from thinking that a patient portal could support adolescents in mental health care, to worrying that it could be detrimental to the treatment. Informants emphasized that the management should facilitate training and support for healthcare providers in using patient portals and telehealth.

**Supplementary Information:**

The online version contains supplementary material available at 10.1186/s12913-023-09156-6.

## Background

Digitalization of health care has opened up for new ways to involve and engage patients, and with this, increased attention has been put on digital patient portals. This is warranted in a time with an increasing demand for health care and where shortages in healthcare workforce have been forecasted [1]. Patient portals have the potential to support adolescents in mental health care and enhance their treatment [2, 3]. Yet, an article from 2017 searched for literature reporting on patient portals in child and adolescent psychiatry or mental health care and found no publications in this area [4]. A repetition of their search in autumn 2022 still did not identify any relevant publications. Thus, there is a knowledge gap on patient portals in adolescent mental health care.

Telehealth is the process of exchanging medical information from one place to another and can be used for remote monitoring of patients, communication and more [1]. Patient portals, which may be an app or a website, are increasingly used for putting telehealth into practice, for example by facilitating online consultations and asynchronous patient-provider communication. Patient portals also include features such as prescription refills, booking appointments and patients viewing information from the electronic health record (EHR) such as laboratory test results and clinical notes [5].

Although there is no research on the use of patient portals for adolescents in mental health care [4], there are some studies on patient portal use for adolescent patients outside mental health care [2, 6–11] and on adults in mental health care [12–20] that can provide valuable insight to the field.

Studies concerning patient portals for adolescent patients outside mental health care reports benefits such as improved patient-provider communication [2, 6, 7] and ease of transition into adult care [7]. In addition, parents’ access to their child’s patient portal can enhance the parents´ understanding of [8] and involvement in the treatment [7, 9]. Both adolescents [7, 10] and parents [2, 7, 8] have reported interest in using patient portals. However, numerous concerns have been raised, such as healthcare providers unintentionally breaking the confidentiality of their younger patients in cases where parents have access to the patient portal [6, 10, 11].

Adults in mental health care report more positive than negative experiences with access to patient portals and EHR-information [14]. The positive experiences include feeling more aware [16] and in control [18] of treatment and increased trust in the healthcare provider [18]. While some healthcare providers report that access to patient portals and EHR information can improve patients´ participation in care [12, 18], it has also been reported that healthcare providers have concerns regarding patient access. For example, some healthcare providers fear that patients may worry when they get access to their clinical notes from their EHR [13, 15, 17, 19], or that they can misinterpret or disagree with the EHR content [14, 18, 20].

Although there are examples of studies on patient portal use for adolescent patients in general and for adults in mental health care, we have found no studies on patient portals for adolescents in mental health care. Hence, the aim of the current study was to explore the views on using patient portals for adolescents in mental health care among persons involved in and/or being affected by the introduction of a new patient portal.

## Methods

A qualitative study with semi-structured individual interviews was conducted between November 2021 and May 2022.

### Setting

The study was conducted in Norway, a Northern-European country with universal health coverage for all inhabitants including primary and specialist healthcare which is funded by payroll contributions and taxes [21]. More precisely the study was performed in Central Norway during the configuration of a new electronic health record (EHR) system for primary and specialist health services, including a patient portal. The current plan is that users of the patient portal can be granted access to information about their health and treatment, a list of vaccinations, communicate with healthcare providers and assess digital solutions such as questionnaires, consultations and booking appointments [22].

In Norway, there is a national patient portal (www.helsenorge.no), which has less functionality than the new patient portal being introduced in Central Norway. However, the regulation for access will be the same, where citizens can access the EHR information in patient portals from the year they become 16 years-old, which is the age of majority under health regulation in Norway [22, 23]. For children under the age of 12 years, parents/guardians have access to most information in their child’s patient portal. However, when the child turns 12 years, the information parents can access becomes restricted and does not include medical records [24]. Moreover, from the age of 12 years, adolescents have the right to decide which healthcare-related information they will share with their parents/guardians [25].

### Informants and recruitment

The inclusion criteria were persons in Central Norway who had either worked as healthcare providers in mental health care or been involved in the development and configuration of the new EHR system including the new patient portal. To capture a diversity of views, we aimed to recruit informants with various roles in the EHR-project (e.g., young representatives from the EHR-project’s user panel representing the service users and patients in the region, management, and subject matter experts representing the field of mental health), and healthcare providers with different professions (e.g., psychologists, nurses, psychiatrists, and other clinical specialists) and from different healthcare settings and areas of Central Norway.

To recruit informants, information about eligible informants was provided by the management in the EHR-project and the department of mental health care at the local hospital. The eligible informants received an invitation letter, an information sheet, and a consent form by email from the first author and were asked to respond if they wanted to participate. Out of the invited 15 persons, one did not have time to participate. A signed consent form was received from all informants prior to their interviews.

### Data collection and interview guide

Data were collected through semi-structured individual interviews. All interviews were conducted by the first author who did not have a prior relationship with any of the informants. Due to pandemic restrictions and geographical distance to some informants, half of the interviews were conducted using the digital platform, Teams. The remaining interviews were conducted through face-to-face meetings at a university in Central Norway. The interviews lasted between 28 minutes and 1 hour and 43 minutes (median 43 minutes). All interviews were audio-recorded and transcribed verbatim by the first author. The analysis was done in parallel to the interviews which were performed until the authors agreed that the data material was sufficient to answer the research question and when no new themes were identified in the last interviews.

A semi-structured interview guide was created for this study based on existing scientific research about patient portal usage in mental health care for adults and for adolescents outside mental health [4, 11, 12, 26], the aim of the study and discussions among the authors. The main questions concerned general views on using patient portals for adolescents in mental health care, views on how or if receiving information about one’s health and health care (e.g., clinical notes in a patient portal) can affect adolescents and their treatment, and views on access to patient portals for parents/guardians and adolescents younger than 16 years of age (see Additional file 1).

### Data analysis

The interviews were transcribed verbatim in the software program NVivo (www.qsrinternational.com/nvivo-qualitative-data-analysis-software/home) by the first author. NVivo was further used for data management and support during the analysis. The analysis was guided by the method of Systematic Text Condensation, a thematic cross-case analysis strategy with four iterative steps: 1) total impression, 2) identifying and sorting meaning units, 3) condensation – from code to meaning and 4) synthesizing – from condensation to descriptions and concepts [27].

At the first step, the first author chose three transcripts that contained large variations in views. These transcripts were read by all authors to gain an overview of the material, and through a discussion among the authors, the preliminary themes were decided on. Secondly, the first author read through all the transcripts and identified meaning units and sorted these into code groups related to the preliminary themes which were discussed, and the process repeated until the final four themes were decided upon. As a third step, the first author wrote condensates representing the voices of the informants based on the meaning units. The condensates were discussed and modified several times among the authors resulting in adjustments of sub-themes and renaming of the themes. As a fourth step, the content of the condensates was synthesised into generalised descriptions and concepts used in the result section, while ensuring that the result still reflected the original context. The translation of the quotes was cross-checked by a native English speaker fluent in Norwegian. To expand and challenge the authors’ perspectives, initial results were presented and debated with other researchers including experts on patient participation and eHealth, and their input was considered in the next phases of the analysis. An outline of the analysis is displayed in an additional file (See Additional file 2).

## Results

In total, 14 informants were included, 10 of whom were females, and 11 of them worked partly or primarily as healthcare providers (Table 1). Some of the informants had several roles. Those affiliated with the EHR-project had been affiliated between 3 months and 7 years. Those who were healthcare providers had between 1,5 and 33 years of work experience.

**Table 1:** Characteristics of the informants

Characteristics	N
**Sex**	
Female	10
Male	4
**Role**	
Healthcare provider in child and adolescent mental health care	11
Affiliated with the EHR-project	5
Young representatives from the EHR-project’s user panel	3
**Age in years**	
< 30	5
30-39	1
40-49	5
> 49	3

All informants showed engagement in the discussion of patient portal use for adolescents in mental health care and had many thoughts on the topic. Yet, many informants also perceived it as a challenging topic with dilemmas linked to the questions of sharing clinical notes with adolescents in mental health care or their parents. Through the analysis, the following four themes were decided upon: “Does access to a patient portal help or harm adolescents?”, “Who decides access?”, “Mostly a political goal” and “Need support and competency”.

### Does access to a patient portal help or harm adolescents?

The informants had different views on whether the use of patient portals would help or harm adolescents in need of mental health care. The views ranged from thinking that providing a digital tool can support adolescents in their treatment, to worrying that adolescents can access information from the EHR that might harm them.

*“We have the medical ethics, right? Never harm. What is meant by “never harming”? Could it also sometimes include withholding information? And here I think that the answer is sometimes yes. Others will think the opposite and say, never harming is giving access to everything” (Healthcare provider).*

Some informants talked about how patient portals can become an additional tool in the treatment where adolescents can receive information and support between consultations and have a more low-threshold communication with the healthcare providers. It was also said that it could encourage adolescents to take on more responsibility and engage them more in their treatment if they had the option to read what was agreed on during the consultation.

*“The areas where there is some resistance, where you need support and a pat on the back to carry out your treatment, it could be anxiety training, when you must carry out uncomfortable things to get better, then it helps that someone sees you. That you experience that someone is with you.” (Healthcare provider and EHR-project affiliated)*

Concerns about whether the use of patient portals in mental health care may harm adolescents were primarily connected to granting access to clinical notes. Several informants spontaneously said that they feared that it could be harmful to the treatment if adolescents read things that made them anxious, without getting the follow-up they needed. Some informants also believed that it could harm the patient-provider relationship and potentially delay the treatment if adolescents read something they disagreed with or experienced as hurtful. Other informants were concerned about whether patients could take charge of their clinical notes, and worried that adolescents uncritically would share sensitive information from their notes on social media. However, some informants also believed that access to clinical notes could help in strengthening the patient’s understanding of their condition and that it could give them more trust in the providers if they could see how the providers were working and what they were writing.

*“I am not as worried for the parents, they can get angry or disagree with my assessment, but I worry for the adolescents, where it can potentially be harmful for them to read. I am worried that this will affect them – their mental health.” (Healthcare provider)*

One topic some informants brought up was whether healthcare providers should change the way they write to avoid harming the adolescents and their treatment. There were different opinions among the informants on whether providers must change their style and write in more patient-friendly language to prevent confusion or worrying the patient. However, most of the informants conveyed the view that providers should continue to write in professional language, yet in a *“warm and respectful way*.” Concerns were also raised regarding whether fewer things would be recorded if the clinical notes were shared with the patients, and some feared that this could be detrimental to the patient.

*“I must inform the patient to a much greater extent on all the assessments I make. What I am a bit afraid of is if I will record fewer assessments. I am a bit afraid that this could be the consequence for healthcare providers across the board. [ … ] If the providers write less, it could potentially result in poorer health care” (Healthcare provider and EHR-project affiliated).*

Some informants believed that it would be easier for providers to share clinical notes with the adolescents if they were honest with the patient during consultation and had a dialogue about the things that would be written in the notes. They also emphasized that providers should tell adolescents that if they chose to use the patient portal or to read their notes, they were always welcome to ask questions. Moreover, some informants believed that providers must introduce and thematize the use of the patient portals for adolescents, both to prevent harm and to clarify if and how it could be used to help the patient.

### Who decides access?

Most informants thought that the providers should have some opportunity to determine which information should be shared and with whom, but several also requested national guidelines and recommendations from their departments on desired practices and rules. Such recommendations and guidelines were wanted to ensure equal practice and to support providers if there was a conflict or a situation where the provider was unsure whether access to clinical information via a patient portal was the best for the patient. However, some, both user representatives and providers, argued that the clinical assessments of the provider must take precedence over such guidelines and added that it was important that deviations from the guidelines happened after a discussion with a colleague.

*“The physician must be able to ask themselves “what suits my patient in this particular case?” So, I mean that if something indicates that one should go against the guidelines, and make another decision, then you should bring it up at a meeting and make the decision together with others.” (Young representative from EHR-projects’ user panel)*

There were different arguments given for why providers should be able to decide which information should be shared and with whom. One area where the informants wanted providers to be able to decide for each patient related to the age at which the patient should get access to their patient portal. They pointed out that it was challenging to set up absolute rules due to the great variation in the maturity of adolescents between 12 and 16 years of age. Whereas some informants emphasized that the patient’s voice should be heard at an early age, others said that it would be better if healthcare providers could adapt whether the adolescent or the parents got access to the patient portal.

Several informants suggested that the providers together with the patient should control which information that was shared with parents. Some explained how parents’ access to the patient portal, including mental health notes, could help adolescents to get more support and understanding at home. However, some also worried that it could make it difficult for the adolescent to be honest during the consultations.

*“Yes, but already now the legislation says that they [young patients] can choose that their parents do not receive information. Depending on age, you should listen to what they say. We must follow the legislation even if technical functionality allows you to read everything. But it is important to agree with the individual patient about what it is okay and not okay to share.” (Healthcare provider).*

### Mostly a political goal

Most informants who were healthcare providers said that sharing clinical notes was a political goal and something the management was interested in, rather than being something that was initiated or needed by healthcare providers. This was not mentioned by any of the young user representatives; however, no informants were asked directly about it. A few informants who were working as healthcare providers argued that, according to them, the EHR primarily belonged to the professionals who used it as a working tool.

*“I do not think I have heard a single provider say: “I think it will be good for the patient to have access to their medical record”. I do not think I have heard anyone say that in psychiatry. The vast majority think that it is not a need we as therapists have. It comes from politicians.” (Healthcare provider and EHR-project affiliated).*

However, some informants argued that increased transparency in health care, including sharing clinical notes, might be challenging yet was a necessary development. One informant described how it was important to *“not let those who are terrified set the limit”.* Similarly, another informant argued that clinical notes should be shared with the patient to achieve *“democratization and sharing of knowledge.”*

*“Healthcare providers are used to being completely autonomous and used to think that they know best. It is a shift in power that lies in it [letting patients get access to clinical notes]. It is not so strange that providers are not so eager to get it. This is also a lifestyle change for doctors. It can lead to you losing a lot, and getting more to do, there are lots of good reasons why health professionals are sceptical.” (Healthcare provider)*

### Need for support and competency

There were different views on who should promote the use of patient portals and access to clinical notes. It was argued that healthcare providers needed to achieve readiness for and competency in using patient portals, and some informants said that the management should help healthcare providers with ways to introduce the patient portal for adolescents.

*“So, it requires willingness and a guided development, and then we [healthcare providers] also need to take control in some ways. That might be the actual job. To get healthcare providers to lead the development and actually do something. We have not come there yet.” (Healthcare provider and EHR-project affiliated)*

Moreover, it was argued that the management should also support and allocate time for healthcare providers to explore ways of using the various features in the patient portal tailored to adolescents in mental health care. Other informants raised the point that the providers needed to lead the change in clinical practice. Some informants believed that the providers would become more engaged in and positive towards patient portals and sharing clinical notes after they had experienced using it as part of the treatment.

## Discussion

The informants’ views on patient portal use in adolescent mental health care varied from thinking it could help adolescents in mental health care to fearing it could harm their treatment. Similarly, parents´ access could increase the support given to adolescents, but also threaten their confidentiality. Most informants thought that healthcare providers should have the autonomy to adjust which information should be shared and with whom. However, several informants also requested national guidelines. Some perceived patient portals as a political decision, and not as a need among healthcare providers, while others described it as a necessary development towards democratization and sharing of knowledge.

### Can support but also harm adolescents

Some informants expected that a patient portal could increase adolescents´ engagement and improve the support given to adolescents during their treatment. This is in line with previous studies from adult mental health care, where access to clinical notes through patient portals improved the adult patients’ control of health care [18], compliance [13], and the patient-provider collaboration and communication [14, 15, 17, 18]. However, other studies from adult mental health care have not found an improved patient-provider relation and collaboration [28–30] or increased patient engagement and involvement [17, 18].

The worries expressed by the informants especially concerned access to clinical notes, and whether such access could lead to harm. Also, studies from adult mental healthcare have reported that providers expected [20, 28] or experienced [17, 18] that the patient’s access to their notes could increase worry [17, 20], confusion [18, 28], or lead to disagreement on the content [20].

Thus, both in the current study and in the literature on patient portal use for both adults in mental health care and adolescent patients outside mental health care, there are arguments pointing to both support and harm. However, this study, as well as some of the referenced literature, illuminates expectations, which are important in the introduction and implementation of patient portals. Still, the actual experience using patient portals can be different. This was shown in a Swedish study where healthcare providers in adult psychiatry were more concerned about potential harm to the patient i.e., worry and confusion, before they started sharing clinical notes [20] than after this was introduced [17]. Similarly, in a study from Northern Norway, many concerns about using patient portals in mental health care were not prevalent among healthcare providers who had shared EHR information via patient portals for 7 years [13]. This points to the importance of not only capturing expectations but also studying the ongoing experiences of using patient portals for adolescents in mental health care.

An additional reason for conducting more research in this area is that adolescents in mental health care can be in an especially vulnerable situation compared to adults. Being an adolescent is a challenging phase in human development, and having mental health problems in this period, adds to this potential vulnerability [31, 32]. Moreover, the changing dynamics between parents and adolescents during this time may cause additional issues that should be considered when sharing EHR information and ensuring the adolescent’s confidentiality. As such, this first study on expectations of patient portal use should be followed by research on what happens in situations when telehealth solutions like patient portals are introduced for adolescents in mental health care.

Some informants believed that national regulations and guidelines from the clinic on patient portal use and sharing EHR information were needed to ensure equal practice between mental healthcare facilities, as well as a support in challenging situations. At the same time, most informants believed that healthcare providers should have the autonomy to make individual decisions based on clinical assessments. However, Norwegian regulations state that information can be withheld from the patient only if *“it is absolutely necessary in order to prevent endangering the patient’s life or serious damage to the patient’s health”* [25]. Hence, the informants’ request for individual assessment of whether access to a patient portal and EHR information will help or harm the individual patient needs to be seen in the light of the patient’s right to access information about their health.

### Access to EHR information

Parents’ access to their child’s patient portal was, on the one hand, perceived by the informants as something that could facilitate better follow-up and support. On the other hand, however, it was perceived as something that could potentially threaten the privacy and confidentiality of the adolescent. This issue has been raised by healthcare providers working with adolescents outside of mental health care [6, 10, 11], and points to the importance of healthcare providers adhering to the needs and integrity of the individual adolescent.

Internationally, great variations exist in regulations on adolescents’ and parents’ access to EHR information [33, 34]. In a study comparing 10 countries, both the age of parental automatic loss of access and the age at which adolescents obtained self-access to the EHR varied from 12 to 18 years and older [33]. Moreover, variations existed on whether there was a gap or an overlap between the adolescent’s access and parental access. Considering the diversity in regulations on adolescent and parental access to EHR information that exists internationally, there is a need for comparative studies providing insight into whether some practices are more suited for adolescents in mental health care than others.

### Digital competency

Several informants said that the management should support healthcare providers in finding ways to introduce and use patient portals. This can be a sensible request, as previous studies have reported that healthcare providers’ introduction and encouragement of a patient portal is an important predictor of patients’ adoption [35, 36]. The type of support that informants asked for also included obtaining readiness for and competencies in engaging with digital solutions such as patient portals. A previous study has suggested digital competency to be important for healthcare providers’ uptake and use of electronic healthcare services [37]. Furthermore, the implementation of a patient portal, including sharing access with patients, did not in itself increase healthcare providers’ digital competency [38]. Hence, as requested by some informants, support from the management with training in patient portal use and forums for collegial discussions appear important for healthcare providers’ adoption and use of patient portals including sharing clinical notes and using telehealth. Based on our results, this should not follow a one-size-fits-all approach, but be tailored to the patient population by e.g., considering the adolescents’ confidentiality and adjustments of access to patient portals and EHR information.

### Strengths and limitations

To the best of our knowledge, this is the first study on patient portals for adolescents in mental health care. Another strength is that the study provides new insights into some of the challenges and potentials related to patient portal use for this group, while also discussing actions to consider when planning, implementing, and using patient portals for adolescents in mental health care.

One limitation is that only views and expectations were studied, and not experiences. Furthermore, no adolescent patients in mental health care were recruited as youth representatives from the user panel were expected to have a broader view as they both had experience as patients receiving health care during childhood and adolescence and with the patient portal being developed. Managers assisted with identifying some of the informants, and this could potentially have affected the recruitment. However, the informants’ anonymity was ensured, and management’s involvement in recruitment did not seem to influence the informant’s statements as they spoke freely about their views. Finally, by recruiting stakeholders from different groups as this was an exploratory study, only a few informants were recruited in each group and the data from each group is thus limited.

## Conclusion

The informants’ views varied from thinking that a patient portal could help adolescents in mental health care, to worrying that it could be detrimental to the treatment. It was argued that regulations and guidelines should be flexible enough to allow some case-by-case adjustments by the healthcare provider, but also ensure equal practice and support in difficult situations. Informants emphasized that the management should facilitate training and support for healthcare providers in using patient portals and telehealth. Research investigating experiences is needed to further understand how patient portals can support adolescents in mental health care.

## Supplementary Information

**Additional file 1. **Interview guide.

**Additional file 2: Table S1.** Outline of the analysis process.

## Data Availability

The data that supports the findings of this study are not openly available due to the regulation of the Regional Ethical Committee, that we must secure the anonymity of the informants, but anonymized transcripts are available from the corresponding author upon reasonable request. The data can be found at the Department of Mental Health at the Norwegian University of Science and Technology in Trondheim, Norway.

## Abbreviation

EHR: Electronic Health Record
