# Supplementary material for: Views on patient portal use for adolescents in mental health care - a qualitative study
Source: BMC Health Serv Res. 2023 Feb 9;23:132. doi: 10.1186/s12913-023-09156-6 (PMC9909909; doi:10.1186/s12913-023-09156-6)
Supplement: Supplementary file 1 — Additional file 1. Interview guide. [file 12913_2023_9156_MOESM1_ESM.docx]

**Interview guide**

1. What are your views on how/if receiving information about one´s health and mental health care (e.g., clinical notes in a patient portal) can affect adolescents and treatment?
2. What are your views on access to patient portals for adolescents (both older and below 16 years old)?
3. What are your views on access to patient portals for parents/guardians and adolescents younger than 16 years of age?
