# Supplementary material for: Views on patient portal use for adolescents in mental health care - a qualitative study
Source: BMC Health Serv Res. 2023 Feb 9;23:132. doi: 10.1186/s12913-023-09156-6 (PMC9909909; doi:10.1186/s12913-023-09156-6)
Supplement: Supplementary file 2 — Additional file 2: Table S1. Outline of the analysis process. [file 12913_2023_9156_MOESM2_ESM.docx]

**Table S1 Outline of the analysis process**

| **Preliminary themes** | **Code groups** | **Final themes** |
| --- | --- | --- |
| A tool in the treatment    Can threaten patient-provider relation  Challenging if parents have access | Increased engagement, and activation of the patient | Does access to a patient portal help or harm? |
|  | Can harm the treatment of adolescents |  |
| Need guidelines to help    Healthcare providers should decide    The difference in the maturity of adolescents | Need regulations and guidelines as support | Who decides access? |
|  | Flexibility to decide for each patient is needed |  |
| Ideology and over-focus on patient rights    Patient-centred care    Increased transparency | Eager management, sceptical providers | Mostly a political goal |
|  | Democratization and sharing of knowledge |  |
| Healthcare providers need support in using patient portals    Change in practice | Support from the management | Need for support and competency |
|  | Healthcare providers need training |  |
